# 

**Published:** 1881-10

**Authors:** 


### CONTENTS.

***ORIGINAL:***

| | PAGE. |
|---|---|
| The Homœopathic Convention of 1881. P. P. Wells, M. D., | 459 |
| The Penn Medical University, | 466 |
| Wanted, | 466 |
| "Gemiasma Verdans," | 466 |
| Fatal Errors. Ad. Lippe, M. D., Philadelphia, | 467 |
| Fluxion Dilutions. Wm. Jefferson Guernsey, M. D., Philadelphia, | 471 |
| "Diphtheria and Bacteria:" A Criticism Criticised. R. R. Gregg, M. D., Buffalo N. Y., | 474 |
| Epigæa: A Proving. C. F. Millspaugh, M. D., Binghamton, N. Y., | 487 |
| Following Pickwick's Advice, | 489 |

***CLINICAL BUREAU:***

| | |
|---|---|
| Intermittent Fever-Cases. E. W. Rushmore, M. D., Plainfield, N. J., | 490 |
| Rheumatism-Cases C. F. Nichols, M. D., Boston, | 491 |

***CLINICAL CASES:***

| | |
|---|---|
| *Petro.* and *Kali*—Post-partum derangements; *Calc.*—Headache; *Bry.*—Cough; *Castoreum*—Abdominal complaints; *Sulph.*—Piles; *Ranun. Scel.*—Corns. E. W. Berridge, M. D., London, | 496 |
| PERISCOPE. W. M. J., | 501 |

***BOOK NOTICES, REVIEWS, ETC.***

| | |
|---|---|
| Notes on Current *Literature,* | 507 |

